# Least Square Regression Method for Estimating Gas Concentration in an Electronic Nose System

**DOI:** 10.3390/s90301678

**Published:** 2009-03-10

**Authors:** Walaa Khalaf, Calogero Pace, Manlio Gaudioso

**Affiliations:** Dipartimento di Elettronica Informatica e Sistemistica, Università della Calabria, 87036 Rende (CS), Italy; E-Mails: cpace@unical.it; gaudioso@deis.unical.it

**Keywords:** Electronic Nose, Support Vector Machine, Least Square Regression, Classification, Concentration Estimation

## Abstract

We describe an Electronic Nose (ENose) system which is able to identify the type of analyte and to estimate its concentration. The system consists of seven sensors, five of them being gas sensors (supplied with different heater voltage values), the remainder being a temperature and a humidity sensor, respectively. To identify a new analyte sample and then to estimate its concentration, we use both some machine learning techniques and the least square regression principle. In fact, we apply two different training models; the first one is based on the Support Vector Machine (SVM) approach and is aimed at teaching the system how to discriminate among different gases, while the second one uses the least squares regression approach to predict the concentration of each type of analyte.

## 1. Introduction

The paper deals with the problems of gas detection and recognition, as well as concentration estimation. The fast evaporation rate and toxic nature of many Volatile Organic Compounds (VOCs) could be dangerous for the health of humans at high concentration levels in air and workplaces, therefore the detection of these compounds has become a serious and important task in many fields. In fact, VOCs are also considered as the main reason for allergic pathologies, lung and skin diseases. Other applications of systems for gas detection are in environmental monitoring, food quality assessment [1], disease diagnosis [2–3], and airport security [4].

There are many research contributions on the design of an electronic nose system based on using tin oxide gas-sensors array in combination with Artificial Neural Networks (ANN) for the identification of the Volatile Organic Compounds (VOC’s) relevant to environmental monitoring, Srivastava [5] used a new data transformation technique based on mean and variance of individual gas-sensor combinations to improve the classification accuracy of a neural network classifier. His simulation results demonstrated that the system was capable of successfully identifying target vapors even under noisy conditions. Simultaneous estimates of many kinds of odor classes and concentrations have been made by Daqi *et al*. [6]; they put the problem in the form of a multi-input/multi-output (MIMO) function approximation problem.

In the literature several different approximation models have been adopted. In particular a multivariate logarithmic regression (MVLR) has been discussed in [7], a quadratic multivariate logarithmic regression (QMVLR) in [8], while a multilayer perceptron (MLP) has been experimented in [4]. Finally, support vector machines (SVM) has been used in [9–11].

We formulate the problem of gas detection and recognition in the form of a two-class or a multi-class classification problem. We perform classification for a given set of analytes. To identify the type of analyte we use the support vector machine (SVM) approach, which was introduced by Vapnik [12] as a classification tool and strongly relies on statistical learning theory. Classification is based on the idea of finding the best separating hyperplane (in terms of classification error and separation margin) of two point-sets in the sample space (which in our case is the Euclidean seven-dimensions vector space, since each sample corresponds to the measures reported by the seven sensors which constitute the core of our system). Our classification approach includes the possibility of adopting kernel transformations within the SVM context, thus allowing calculation of the inner products directly in the feature space without explicitly applying the mapping [13].

As previously mentioned, we adopt a multi-sensor scheme and useful information is gathered by combining the outputs of the different sensors. In fact, in general the use of just one sensor does not allow identification of a gas, as the same sensor output may correspond to different concentrations of many different analytes. On the other hand, by combining the information coming from several sensors of diverse types under different heater voltages values we are able to identify the gas and to estimate its concentration.

The paper is organized as follows. In Section 2 we describe our Electronic Nose (ENose), while Section 3 gives a brief overview of the SVM approach. Section 4 is devoted to the description of our experiments involving five different types of analytes (acetone, benzene, ethanol, isopropanol, and methanol). Finally the conclusions are drawn in Section 5.

## 2. Electronic Nose

**Figure 1.**
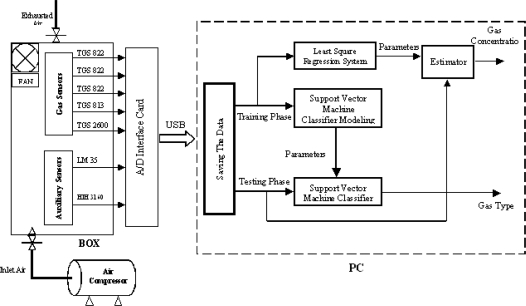
Block diagram of the system.

**Figure 2.**
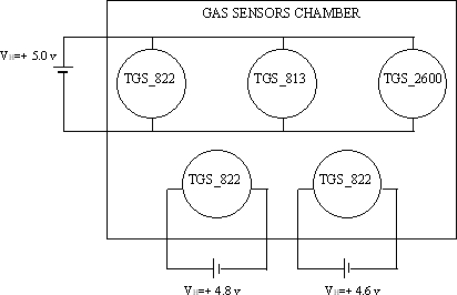
Block diagram of the sensors heater voltage supplies.

An electronic nose is an array of gas sensors, whose response constitutes an odor pattern [14]. A single sensor in the array should not be highly specific in its response but should respond to a broad range of compounds, so that different patterns are expected to be related to different odors. To achieve high recognition rates, several sensors with different selectivity patterns are used and pattern recognition techniques must be coupled with the sensor array [10]. Our system (Figure 1) consists of five different types of gas sensors supplied with different heater voltages to improve the selectivity and the sensitivity of the sensors which are from the TGS class of FIGARO USA, Inc. The sensing element is a tin dioxide (*SnO*_2_) semiconductor layer. In particular three of them are of TGS-822 type, each one being supplied with a different heater voltage (5.0 V, 4.8 V, and 4.6 V, respectively, see Figure 2), one of the TGS-813 type, and the last one is of the TGS-2600 type. Because the gas sensor response is heavily affected by environmental changes, two auxiliary sensors are used for the temperature (LM-35 sensor from National Semiconductor Corporation), and for the humidity (HIH-3610 sensor from Honeywell).

The gas sensors and the auxiliary sensors are put in a box of 3000 cm^3^ internal volume. Inside the box we put a fan to let the solvent drops evaporate easily. All sensors are connected to a multifunction board (NI DAQPad-6015), which is used in our system as an interface between the box and the PC. The National Instruments DAQPad-6015 multifunction data acquisition (DAQ) device provides plug-and-play connectivity via USB for acquiring, generating, and logging data; it gives 16-bit accuracy at up to 200 kS/s, and allows 16 analog inputs, 8 digital I/O, two analog outputs, and two counter/timers. NI DAQPad-6015 includes NI-DAQmx measurement services software, which can be quickly configured and allows us to take measurements with our DAQ device. In addition NI-DAQmx provides an interface to our LabWindows/CVI [15] running on our Pentium 4 type PC.

The integrated LabWindows/CVI environment features code generation tools and prototyping utilities for fast and easy C code development. It offers a unique, interactive ANSI C approach that delivers access to the full power of C Language. Because LabWindows/CVI is a programming environment for developing measurement applications, it includes a large set of run-time libraries for instrument control, data acquisition, analysis, and user interface. It also contains many features that make developing measurement applications much easier than in traditional C language environments.

For support vector machine (SVM) training and testing in multi-class classification we use LIBSVM-2.82 package [16]. LIBSVM-2.82 uses the one-against-one approach [17] in which, given **k** distinct classes, **k(k −1)/2** binary classifiers are constructed, each one considering data from two different classes. LIBSVM provides a parameter selection tool for using different kernels and allows cross validation. For median-sized problems, cross validation might be the most reliable way for parameter selection. First, the training data is partitioned into several folds. Sequentially a fold is considered as the validation set and the rest are for training. The average of accuracy on predicting the validation sets is the cross validation accuracy [18]. In particular the leave-one-out cross validation scheme consists of defining folds which are singletons, i.e. each of them is constituted by just one sample.

## 3. Support Vector Machine (SVM)

Support vector machines (SVMs) are a set of related supervised learning methods used for classification and regression of multi dimensional data sets [19, 14]. They belong to the family of generalized linear classifiers. This family of classifiers has both the abilities of minimizing the empirical classification error and maximizing the geometric margin. In fact a SVM is also known as maximum margin classifier [9]. In this section we summarize the main features of SVM. Detailed surveys can be found in [3, 14, 20–21]. SVM looks for a separating hyperplane between the two data sets. The equation of such hyperplane is defined by:

(1) $$f(x)=w^{T}\;x+b=0$$

where **w** is the weight vector which defines a direction perpendicular to the hyperplane, **x** is the input data point, and ***b*** is the bias value (scalar), for a proper normalization. The margin is equal to ||**w**||^−1^. Therefore maximizing the margin is equivalent to minimizing ||**w**||. The advantage of this maximum margin criterion is both robustness against noise and uniqueness of the solution.

In many practical cases the data are not linearly separable, then the hyperplane tries to both maximize the margin and minimize the sum of classification errors at the same time. The error *ξ_i_* of a point (*x_i_*,*y_i_*) (*y_i_* ∈ {−1,+1} represents the class membership) with respect to a target margin *γ* and for a hyperplane defined by *f* is:

(2) $$\xi_{i}=\xi ((x_{i},\;y_{i}),\;f(x_{i}),\;\gamma )=\text{max}(0,\gamma -y_{i}\;f(x_{i}))$$

where *ξ_i_* is called the margin slack variable which measures how much a point fails to have margin. If *y_i_* and *f*(x*_i_*) have different signs the point x*_i_* is misclassified because

(3) $$\xi_{i}>\gamma >0$$

The error *ξ_i_* is greater than zero if the point x*_i_* is correctly classified but with margin smaller than *γ*.

(4) $$\gamma >\xi_{i}>0$$

Finally, the more x*_i_* falls in the wrong region, i.e. satisfies equation 3, the bigger is the error. The cost function to be minimized is:

(5) $$\frac{1}{2}{||w||}^{2}+C\sum_{i}\xi_{i}$$

where *C* is a positive constant, which determines the trade off between accuracy in classification and margin width [20–21]. Therefore, this constant can be regarded as a *regularization parameter*. When *C* has a small value, the optimal separating hyperplane tends to maximize the distance with respect to the closest point, while for large values of *C*, the optimal separating hyperplane tends to minimize the number of non-correctly classified points.

If the original patterns are not linearly separable, they can be mapped by means of appropriate kernel functions to a higher dimensional space called *feature space*. A linear separation in the feature space corresponds to a non-linear separation in the original input space [11]. Kernels are a special class of functions that permit the inner products to be calculated directly in the feature space, without explicitly applying the mapping. The family of kernel functions adopted in machine learning range from simple linear and polynomial mappings to sigmoid and radial basis functions [22]. In this paper linear kernel is used.

## 4. Experiments and Results

In our experiments we used five different types of volatile species with different concentrations. They are acetone, methanol, ethanol, benzene, and isopropanol. The data set for these volatile species is made up of samples in R^7^ space where each sample correspond to the outputs of the gas and auxiliary sensors.

### 4.1. Samples Preparation

Our box contains the PCB (Printed Circuit Board) where we fixed two different types of sensors, i.e. gas sensors and auxiliary sensors. It also contains a fan for circulating the analyte inside during the test. The system encompasses one input for inlet air coming from an air compressor which has been used to clean the box and the gas sensors after each test. One output is used for the exhaust air. The inner dimensions of the box are 22 cm length, 14.5 cm width, and 10 cm height, while the effective volume is 3,000 cm^3^. The amount of volatile compounds needed to create the desired concentration in the sensor chamber (our box) was introduced in the liquid phase using a high-precision liquid chromatography syringe. Since temperature, pressure and volume were known, the liquid needed to create the desired concentration of volatile species inside the box could be calculated using the ideal gas theory, as we explain below. The analyte concentration versus analyte volume injected is shown in Table 1.

A syringe of 10 μL is used for injecting the test volatile compounds. We take methanol as an example for calculating the ppm (parts-per-million) for each compound. Methanol has a molecular weight *MW* = 32.04 g/mol and density ρ = 0.7918 g/cm^3^. The volume of the box is 3,000 cm^3^; therefore, for example, to get 100 ppm inside the box, from Table 1, we used 0.3 cm^3^ of methanol.

The density of methanol is

(6) $$\partial =\frac{P\times \mathrm{MW}}{R\times T}$$

Where:

∂ = the density of the gas of Methanol in g/L,
*P* = the Standard Atmospheric Pressure (in atm) is used as a reference for gas densities and volumes (equal 1 atm),
*MW* = Molecular Weight in g/mol,
*R* = universal gas constant in atm/mol.K (equal 0.0821 atm/mol.K),
*T* = temperature in Kelvin (*T_K_* = *T_C_* + 273.15).

As a result we get d = 1.33 g/L.

(7) $$\text{Mass}=\nu_{\text{gas}}*\partial =\nu_{\text{liq}}*\rho$$

where *v_gas_* is the volume occupied by the gas of methanol which is equal to 0.3*10^−3^ *l*, ∂ is the density of the gas of Methanol as calculated before, ρ is the constant density of methanol, therefore; *v*_liq_ = (*v_gas_* × ∂) / ρ ⇒ *v*_liq_ = (0.3 * 10^−3^ * 1.33) / 0.7918, the volume (*v*_liq_) is 0.503*10^−6^ *l* which provides 100 ppm of methanol. This means that if we want to get 100 ppm of methanol we must put 0.503 μL of liquid methanol in the box by using the syringe. Table 2 shows different concentrations of Methanol (in ppm) versus its quantities (in μL).

### 4.2. Results

In the first analysis, we used a SVM with linear kernel, and we applied a multi-class classification by using the LIBSVM-2.82 package [16]. The optimal regularization parameter *C* was tuned experimentally by minimizing the leave-one-out cross-validation error over the training set.

In fact the program was trained as many times as the number of samples, each time leaving out one sample from training set, and considering such omitted sample as a testing sample check the classification correctness. The classification correctness rate is the average ratio of the number of samples correctly classified and the total number of samples. The results are shown in Table 3 for different values of *C*. We used 22 concentration samples for acetone, 22 for benzene, 20 for ethanol, 23 for isopropanol, and 21 for methanol. For each concentration the experiment was repeated twice, thus a total number of 216 classification calculations was performed.. By using linear kernel we got 100.00% classification correctness rate for C = 1,000 adopting a leave-one-out cross-validation scheme. We remark that such results are better than those obtained by supplying all sensors by the same heater voltage (in such case, in fact, the best classification correctness rate was 94.74%).

Once the classification process has been completed, the next step is to estimate the concentration of the classified analyte. To this aim, we use the least square regression approach. We build an approximation of the response (sensor resistance versus analyte concentration) for each sensor and each analyte. Then we use this approximation to find the concentration for each analyte type.

For sintered *SnO*_2_ gas sensor, the concentration dependence of the response to a simple analyte exposure is nonlinear and can be described by a power law of the form [23]

(8) $$R={\delta c}^{\omega }$$

where *R* is the sensor resistance, *δ* a constant, *c* the concentration of the analyte and ω an index that lies between 0.3 and 1.0. We applied this equation on all sensors for each analyte. The values of *δ* ‘s and ω ‘s, are calculated as follows:

(9) $$\begin{array}{l} \Omega =\frac{n\sum_{i=1}^{n}(\text{ln}\;c_{i}\;\text{ln}\;R_{i})-\sum_{i=1}^{n}\left(\text{ln}\;c_{i})\sum_{i=1}^{n}(\text{ln}\;R_{i}\right)}{n\sum_{i=1}^{n}{\left(\text{ln}\;c_{i}\right)}^{2}-{\left(\sum_{i=1}^{n}\text{ln}\;c_{i}\right)}^{2}}\\ \Delta =\frac{\sum_{i=1}^{n}\left(\text{ln}\;R_{i}\right)-\Omega \sum_{i=1}^{n}\left(\text{ln}\;c_{i}\right)}{n} \end{array}$$

where *ω* ≡ Ω, *δ* ≡ exp(Δ) and *n* is the number of samples, which are indexed by *i.*

**Figure 3.**
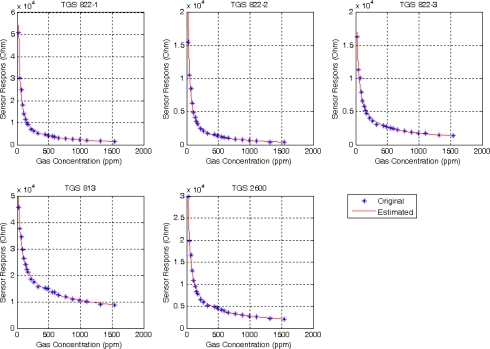
Acetone concentrations vs. sensor resistance for each sensor type.

Figure 3 shows, as an example, the original concentrations with respect to their sensor resistances, as well as the estimated curve for the analyte acetone. We have five curves, one for each sensor.

The optimal estimate of the concentration is in our model a combination of the outputs of the diverse sensors. We have adopted the least square regression model to find the optimal weights on the basis of the experimental data. We come out in our experiments with five measures for each analyte sample. The weights α’s are obtained by solving the following minimization problem :

(10) $$\underset{\alpha_{i},...,\alpha_{m}}{\text{min}}\sum_{l=1}^{n}{\left({\bar{c}}^{\left(l\right)}-\sum_{i=1}^{M}\alpha_{i}\;c_{i}^{\left(l\right)}\right)}^{2}$$

where *n* is the number of analyte samples, *c̄* is the true concentration, *M* is the number of sensors (in our case *M* = 5), *c* the concentrations that have been previously calculated (from equation 8). Tables 4–8 show the real concentrations with respect to the results of the proposed method. For comparison purposes we add in the table also the results obtained by simply averaging the outcomes provided by the five sensors.

Finally we considered (Table 9) the correlation coefficient (*C.C*) as a measure for the estimation accuracy [8]. The correlation coefficient is a number between 0.0 and 1.0. If there is no relationship between the predicted values and the actual values the correlation coefficient is 0.0 or very low (the predicted values are no better than random numbers). As the strength of the relationship between the predicted values and actual values increases so does the correlation coefficient. A perfect fit gives a coefficient of 1.0. Thus the higher correlation coefficient (near to 1.0) the better is the regressor [7]. The correlation coefficient is calculated as follows:

(11) $$C.C=\frac{\sum_{i=1}^{n}X_{i}\;{\hat{X}}_{i}-\frac{\sum_{i=1}^{n}X_{i}\sum_{i=1}^{n}{\hat{X}}_{i}}{n}}{\sqrt{(\sum_{i=1}^{n}X_{i}^{2}-\frac{{\left(\sum_{i=1}^{n}X_{i}\right)}^{2}}{n})\;(\sum_{i=1}^{n}{\hat{X}}_{i}^{2}-\frac{{\left(\sum_{i=1}^{n}{\hat{X}}_{i}\right)}^{2}}{n})}}$$

where *C.C* is the correlation coefficient, *X* are the actual values, *X̂* are the predicted values, and *n* is the number of data points.

## 5. Conclusions

The results demonstrate that our system has the ability to identify the type of analyte and then estimate its concentration. The best correctness rate was 100.00%. Also the values obtained in terms of concentration estimates appear quite satisfactory. Supplying three similar sensors (TGS-822) with different heater voltages, improved the performance of the system. Future work will be devoted to identify binary mixture of gases and then to estimate the concentration of each component.

## Figures and Tables

**Table 1.** Analyte concentration vs. analyte volume.

**Analyte Concentration (ppm)**	**Volume of Pure Analyte (cm^3^)**
10	0.03
50	0.15
100	0.30
200	0.60
400	1.20
800	2.40
1,000	3.00
2,000	6.00

**Table 2.** Methanol concentration vs. methanol quantity.

**Methanol Concentration (ppm)**	**Methanol quantity (μL)**
40	0.2
100	0.5
200	1.0
400	2.0
800	4.0
1,000	5.0
1,400	7.0
2,000	10.0

**Table 3.** Multiple C values with linear kernel.

**C values**	**Classification Correctness Rate %**
10	91.24
50	96.31
100	96.77
500	98.62
800	99.08
1,000	100.00
2,000	99.54

**Table 4.** Experimental results of acetone.

**Real data**	**Our Method**	**Average Method**	**% Absolute Error of our Method**	**% Absolute Error of Average Method**
22	22.68	25.11	3.110	14.136
44	43.85	43.75	0.339	0.574
66	55.79	55.17	15.470	16.404
88	82.23	80.45	6.554	8.579
110	110.92	108.18	0.841	1.651
132	137.36	133.22	4.058	0.928
154	165.54	160.75	7.492	4.383
176	189.89	180.88	7.891	2.775
220	252.73	244.37	14.878	11.079
264	300.03	287.54	13.647	8.918
330	379.21	366.94	14.913	11.194
440	428.31	407.13	2.657	7.470
484	474.41	443.42	1.981	8.384
550	533.80	520.15	2.945	5.426
594	585.78	552.30	1.383	7.020
660	649.93	644.15	1.526	2.401
770	761.83	753.86	1.060	2.095
880	873.91	880.84	0.692	0.095
990	997.66	1,,010.21	0.774	2.042
1,100	1,057.31	1096.75	3.881	0.296
1,320	1,353.41	1,425.29	2.531	7.977
1,540	1,534.37	1,609.86	0.365	4.536

**Table 5.** Experimental results of methanol.

**Real data**	**Our Method**	**Average Method**	**% Absolute Error of our Method**	**% Absolute Error of Average Method**
40	41.09	38.76	2.7325	3.1075
80	76.49	70.86	4.3903	11.4300
120	118.94	125.38	0.8866	4.4875
160	164.71	162.21	2.9463	1.3839
200	193.07	196.57	3.4661	1.7135
240	237.82	245.19	0.9100	2.1653
280	273.81	284.39	2.2116	1.5684
320	307.61	329.55	3.8700	2.9860
360	367.57	380.85	2.1027	5.7927
400	408.58	439.90	2.1463	9.9751
480	481.48	5060.53	0.3087	5.4278
600	598.60	620.04	0.2324	3.3397
720	689.76	678.69	4.2004	5.7369
800	819.69	794.98	2.4616	0.6268
960	983.12	990.82	2.4083	3.2107
1,080	1,083.01	1,089.45	0.2790	0.8751
1,200	1,178.88	1,178.12	1.7597	1.8232
1,400	1,395.61	1,364.58	0.3136	2.5300
1,600	1,597.26	1,547.35	0.1711	3.2908
1,800	1,810.56	1,761.23	0.5868	2.1540
2,000	1,997.90	1,909.19	0.1047	4.5406

**Table 6.** Experimental results of ethanol.

**Real data**	**Our Method**	**Average Method**	**% Absolute Error of our Method**	**% Absolute Error of Average Method**
27	23.84	24.47	11.6921	9.3663
54	55.01	51.43	1.8721	4.7533
81	90.55	84.19	11.7858	3.9464
108	106.07	99.83	1.7893	7.5666
135	143.28	135.36	6.1326	0.2645
162	172.99	164.72	6.7826	1.6814
189	198.07	201.59	4.8000	6.6625
216	223.99	237.61	3.6991	10.0069
243	242.01	264.74	0.4110	8.9485
270	288.60	283.36	6.8887	4.9494
324	336.74	332.17	3.9314	2.5230
405	422.82	428.93	4.4003	5.9088
459	455.81	484.86	0.6948	5.6352
540	527.63	576.41	2.2900	6.7433
675	661.31	622.77	2.0285	7.7381
810	820.18	747.14	1.2567	7.7607
945	928.10	887.55	1.7881	6.0792
1,080	1,049.95	1,002.22	2.7819	7.2019
1,350	1,373.04	1,326.66	1.7067	1.7287

**Table 7.** Experimental results of benzene.

**Real data**	**Our Method**	**Average Method**	**% Absolute Error of our Method**	**% Absolute Error of Average Method**
18	15.14	16.21	15.8811	9.9555
36	36.63	35.09	1.7508	2.5043
54	56.52	55.47	4.6647	2.7224
72	75.08	74.35	4.2835	3.2601
90	96.30	96.08	6.9992	6.7518
108	115.37	116.10	6.8290	7.5011
126	129.59	130.91	2.8540	3.9012
144	150.63	154.68	4.6053	7.4186
162	166.19	170.06	2.5878	4.9739
180	185.53	187.88	3.0753	4.3775
234	248.06	246.47	6.0083	5.3295
270	274.97	276.51	1.8425	2.4126
324	325.56	326.13	0.4829	0.6590
360	353.29	356.88	1.8619	0.8661
414	415.01	407.07	0.2453	1.6730
468	449.41	447.26	3.9718	4.4310
540	514.60	503.90	4.7034	6.6841
630	637.34	641.77	1.1648	1.8692
720	738.37	726.53	2.5518	0.9075
810	806.19	794.96	0.4706	1.8567
900	904.35	860.47	0.4839	4.3921
1,080	1,074.29	1,072.02	0.5286	0.7390

**Table 8.** Experimental results of isopropanol.

**Real data**	**Our Method**	**Average Method**	**% Absolute Error of our Method**	**% Absolute Error of Average Method**
21	17.85	16.81	14.9841	19.9374
42	42.39	42.65	0.9247	1.5425
63	66.31	67.19	5.2505	6.6589
84	94.75	93.65	12.8003	11.4851
105	112.92	113.76	7.5438	8.3477
126	130.53	131.09	3.5948	4.0371
147	160.04	157.52	8.8746	7.1591
168	173.03	171.97	2.9935	2.3634
189	197.58	199.13	4.5414	5.3587
210	211.78	212.78	0.8498	1.3222
252	255.39	255.41	1.3471	1.3522
294	298.80	292.86	1.6342	0.3873
357	348.83	343.44	2.2893	3.7973
420	401.99	407.81	4.2879	2.9030
483	482.31	470.84	0.1420	2.5169
567	567.14	548.54	0.0257	3.2557
630	648.76	626.99	2.9784	0.4781
735	720.55	695.12	1.9660	5.4257
840	833.88	818.79	0.7283	2.5243
945	934.93	915.13	1.0650	3.1612
1,050	1,071.76	1,068.29	2.0723	1.7416
1,260	1,251.77	1,260.44	0.6530	0.0350

**Table 9.** Correlation Coefficient (C.C) value for each analyte.

**Analyte Type**	**C.C from the new method**	**C.C from the method of average**	**C.C from SVM regression method**
Acetone	0.998930	0.997757	0.982431
Benzene	0.999535	0.999196	0.989445
Ethanol	0.999394	0.997515	0.974048
Isopropanol	0.999629	0.999322	0.985179
Methanol	0.999803	0.999251	0.973584

## References

[1] Zhang H., Chang M., Wang J., Ye S. (2008). Evaluation of Peach quality Indices Using an Electronic Nose by MLR, QPST, and BP Network. Sens. Actuat. B.

[2] Casalinuovo I., Pierro D. (2006). Application of Electronic Noses for Disease Diagnosis and Food Spoilage Detection. Sensors.

[3] Gardner J.W., Shin H., Hines E. (2000). An Electronic Nose System to Diagnose Illness. Sens. Actuat. B.

[4] Lee D., Lee D., Ban S., Lee M., Kim Y. (2002). SnO_2_ Gas Sensing Array for Combustible and Explosive Gas Leakage Recognition. IEEE Sensors J.

[5] Srivastava A.K. (2003). Detection of Volatile Organic Compounds (VOCs) Using SnO_2_ Gas-Sensor Array and Artificial Neural Network. Sens. Actuat. B.

[6] Daqi G., Wei C. (2007). Simultaneous Estimation of Odor Classes and Concentrations Using an Electronic Nose with Function Approximation Model Ensembles. Sens. Actuat. B.

[7] Cohen J., Cohen P., West S.G., Aiken L.S. (2003). Applied Multiple Regression/Correlation Analysis for the Behavioural Sciences.

[8] Penza M., Cassano G., Tortorella F. (2002). Identification and Quantification of Individual Volatile Organic Compounds in a Binary Mixture by Saw Multisensor Array and Pattern Recognition Analysis. Meas. Sci. Technol.

[9] Distante C., Ancona N., Siciliano P. (2003). Support Vector Machines for Olfactory Signals Recognition. Sens. Actuat. B.

[10] Pardo M., Sberveglieri G. (2005). Classification of Electronic Nose Data with Support Vector Machines. Sens. Actuat. B.

[11] Wang X., Zhabg H., Zhang C. Signals Recognition of Electronic Nose Based on Support Vector Machines.

[12] Vapnik V.N. (1998). Statistical Learning Theory.

[13] Shawe-Taylor J, Cristianini N. (2004). Kernel Methods for Pattern Analysis.

[14] Pearce T.C., Schiffman S.S., Nagle H.T., Gardner J.W. (2003). Handbook of Machine Olfaction: Electronic Nose Technology.

[15] National Instruments http://www.ni.com/lwcvi/.

[16] Chang C., Lin C. (2001). Libsvm: A Library for Support Vector Machines. http://www.csie.ntu.edu.tw/cjlin/libsvm.

[17] Knerr S., Personnaz L., Dreyfus G., Fogelman J. (1990). Single-Layer Learning Revisited: a Stepwise Procedure for Building and Training a Neural Network. Neurocomputing: Algorithms, Architectures and Applications.

[18] Gallant S.I. (1993). Neural Network Learning and Expert Systems.

[19] Gutierrez-Osuna R. (2003). Pattern Analysis for Machine Olfaction: A Review. IEEE Sensors J.

[20] Burges C. (1998). A Tutorial on Support Vector Machines for Pattern Recognition. Data Min. Knowl. Discov.

[21] Cristianini N., Shawe-Taylor J. (2000). An Introduction to Support Vector Machines and Other Kernel-based Learning Methods.

[22] Mouller K., Mika S., Ratsch G., Tsuda K., Scholkopf B. (2001). An Introduction to Kernel-Based Learning Algorithms. IEEE Trans. Neural Networks.

[23] Bartlett P., Gardner J.W. (1992). Odour Sensors for an Electronic Nose, in Sensors and Sensory Systems for an Electronic Nose. NATO ASI Ser. Appl. Sci.

